# TRIM21 Promotes Rabies Virus Production by Degrading IRF7 through Ubiquitination

**DOI:** 10.3390/ijms241310892

**Published:** 2023-06-30

**Authors:** Boyue Zhang, Ting Cai, Hongling He, Xuezhe Huang, Guie Chen, Yanqin Lai, Yongwen Luo, Shile Huang, Jun Luo, Xiaofeng Guo

**Affiliations:** 1 College of Veterinary Medicine, South China Agricultural University, Guangzhou 510000, China; zhangboyue163@163.com (B.Z.); 18819773609@163.com (T.C.); 13610205214@163.com (H.H.); scauhxz@163.com (X.H.); guiechan@163.com (G.C.); yanqinlai97@163.com (Y.L.); ywluo@scau.edu.cn (Y.L.); 2 Department of Biochemistry and Molecular Biology, Louisiana State University Health Sciences Center, 1501 Kings Highway, Shreveport, LA 71130-3932, USA; shuan1@lsuhsc.edu; 3 Feist-Weiller Cancer Center, Louisiana State University Health Sciences Center, Shreveport, LA 71130-3932, USA

**Keywords:** rabies virus, interferon, IRF7, TRIM21, ubiquitination

## Abstract

Rabies, a highly fatal zoonotic disease, is a significant global public health threat. Currently, the pathogenic mechanism of rabies has not been fully elucidated, and no effective treatment for rabies is available. Increasing evidence shows that the tripartite-motif protein (TRIM) family of proteins participates in the host’s regulation of viral replication. Studies have demonstrated the upregulated expression of tripartite-motif protein 21 (TRIM21) in the brain tissue of mice infected with the rabies virus. Related studies have shown that TRIM21 knockdown inhibits RABV replication, while overexpression of TRIM21 exerted the opposite effect. Knockdown of interferon-alpha and interferon-beta modulates the inhibition of RABV replication caused by TRIM21 knockdown and promotes the replication of the virus. Furthermore, our previous study revealed that TRIM21 regulates the secretion of type I interferon during RABV infection by targeting interferon regulatory factor 7 (IRF7). IRF7 knockdown reduced the inhibition of RABV replication caused by the knockdown of TRIM21 and promoted viral replication. TRIM21 regulates RABV replication via the IRF7-IFN axis. Our study identified TRIM21 as a novel host factor required by RABV for replication. Thus, TRIM21 is a potential target for rabies treatment or management.

## 1. Introduction

Rabies is a zoonotic infectious disease caused by the rabies virus (RABV), with a dysfunctional central nervous system (CNS) being the main manifestation. The mortality rate of rabies is almost 100% without effective treatment. There are approximately 59,000 people worldwide who die from rabies annually [1]. The rabies virus belongs to the Mononegavirales order in the Rhabdoviridae family and Lyssavirus genus. The RABV genome is about 12 kb in size and comprises five genes encoding a nucleoprotein (N), phosphoprotein (P), matrix protein (M), glycoprotein (G), and the RNA-dependent RNA polymerase (L). All the structural proteins of RABV are critical for virus production [2,3,4]. At present, several facts about the virus, some of which could be exploited to treat or manage rabies, are still unknown. Therefore, it is necessary to identify more host factors that regulate RABV to elucidate the underlying mechanisms of viral infection and subsequently to provide new therapeutic strategies against RABV.

Innate immunity is the host’s first line of defence against viral infection. Type I interferon (IFNα/IFNβ), the main effector of innate immunity, plays an important antiviral role [5,6,7,8]. Studies have shown that the N, P, and M proteins of the rabies virus are involved in the immune escape of the virus [9,10,11]. The host recognizes RABV through several pattern recognition receptors, including Retinoic acid-inducible gene 1 (RIG-I), Melanoma differentiation-associated protein 5 (MDA5), and Toll-like [12,13,14,15]. Thereafter, activated mitochondrial antiviral signaling (MAVS) adapters promote the recruitment of kinases, including TANK-binding kinase 1 (TBK1) and IKK, which activate multiple pathways, including interferon regulatory factor (IRF) 3, IRF7, and NF-κB. IRF3 is expressed early in infection but is mostly degraded later in the viral infection stages. IRF7 expression is usually low but increases markedly after viral infection. Studies have shown that IRF7 expression is responsible for the induction of interferon-I (IFN-I) secretion in the late stages of viral infection [16,17,18]. Studies have identified IRF7 as the primary regulator of IFN-I-dependent responses, and IRF7 knockout increased the vulnerability of mice to viral infection [19]. Previous studies have found that IRF7 plays an important regulatory role in the process of RABV infection and inhibits virus replication [20]. However, factors involved in regulating IRF7 expression during RABV infection are still unknown.

Different types of post-translational modifications (PTMs) are critically involved in the control of the immune system. Additionally, PTMs regulate protein activity, stability, folding, localization, and interaction with other molecules. PTMs enhance several antiviral immune responses, such as phosphorylation, ubiquitination, dephosphorylation, and deubiquitination [21,22,23,24]. For example, IRF7 expression is tightly regulated by various PTMs [25]. Transactivation response element RNA-binding protein 2(TARBP2) suppresses IRF7 activation by inhibiting TRAF6-mediated K63-linked IRF7 ubiquitination, which is a prerequisite for IRF7 phosphorylation [8,26]. TRIM8 protects phosphorylated IRF7 from proteasomal degradation in an E3 ubiquitin (Ub) ligase-independent manner [27]. In addition, E3 Ub ligase RAUL inhibits IFN-I production by directly catalyzing the lysine 48-linked ubiquitination of IRF7 and IRF3 [28]. This, in turn, promotes protein degradation, while E3 Ub ligase Pellino 3 regulates TLR3 and viral infection-mediated IFN-I expression by targeting the IRF7 pathway [29,30]. Furthermore, the E3 ligase TRIM28 interacts with IRF7 and increases its SUMOylation (another type of PTM), inhibiting IRF7 activity and IFN-mediated antiviral responses [31]. After influenza A virus infection, degradation of IRF7 by TRIM21 ubiquitination inhibit IFN-I activation [32].

To identify additional host factors that can regulate RABV infection, transcriptomic analysis of mouse brains infected with RABV was performed. We found that several TRIM proteins, especially TRIM21, were significantly differentially expressed in mouse brain tissue after RABV infection. This study focused on the role of TRIM21 in RABV infection. Our results showed that TRIM21 promotes RABV infection by ubiquitinating and degrading IRF7, inhibiting the type I IFN response.

## 2. Results

### 2.1. The Infection of RABV Results in Increased Expression of TRIM21 in NA Cells

**Figure 1:**
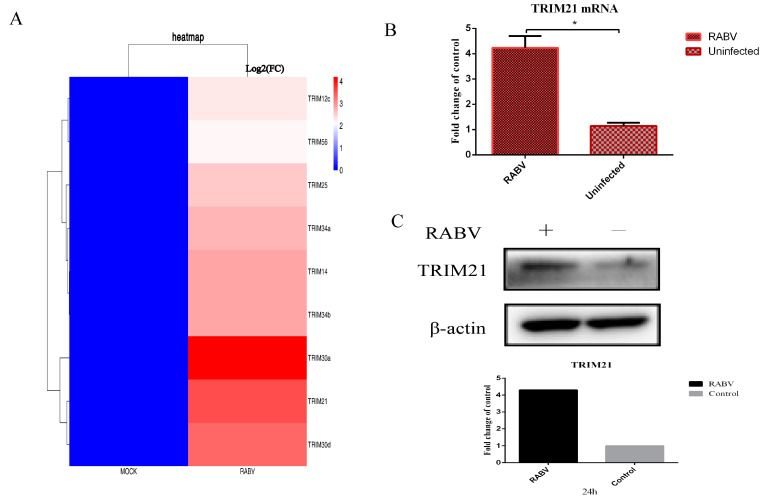
RABV infection upregulates the expression of TRIM21. (**A**) The RNA-seq analysis of TRIM protein-associated host molecules that were differentially expressed (*p* < 0.01) after RABV infection compared to non-infected NA cells at 24 hpi. (**B**,**C**) NA cells were infected with RABV at an MOI of 0.5 for 24 h. Total RNAs were isolated from RABV infected or uninfected NA cells and subjected to qRT-PCR analysis to determine the mRNA levels of TRIM21 (**B**). Data are presented as mean ± SD; *, *p* < 0.05. (**C**). The total protein of treated cells was harvested to determine the protein levels of TRIM21 by Western blotting with anti-TRIM21 antibody. Grayscale analysis of Western blot images using ImageJ.

To systematically profile the potential host factors that respond to RABV, the differentially expressed genes were analyzed based on the data of RNA-seq for mouse brain tissue infected by RABV. The results showed that several ubiquitin associated proteins, particularly some TRIM proteins, were significantly upregulated or downregulated upon RABV infection compared with non-infection (*p* < 0.01) (Figure 1A). To confirm changes in these transcripts, NA cells were infected with RABV at an MOI of 0.5 for 24 h. RT-qPCR and Western blotting were used to verify the levels of mRNA and proteins. We found that RABV infection significantly increased the mRNA (Figure 1B) and protein levels of TRIM21 (Figure 1C). The results suggest that RABV infection increases the expression of TRIM21.

### 2.2. TRIM21 Positively Regulates RABV Replication

**Figure 2:**
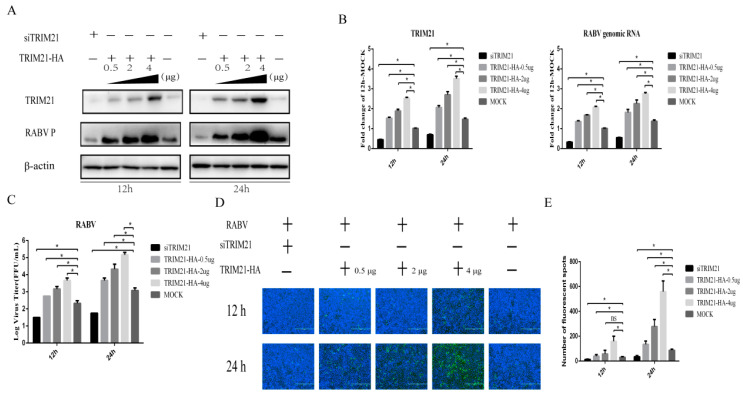
TRIM21 positively regulates the replication of RABV. NA cells were transfected with a pCAGGS-HA-TRIM21 plasmid at different concentrations (0.5 μg, 2 μg, 4 μg) or TRIM21 siRNA for 24 h followed by infection with RABV at an MOI of 0.5 for another 12 h or 24 h. No infection (Mock) was performed as the control group. An empty vector plasmid (pCAGGS-HA) or a non-targeting control siRNA (NC) served as the control. (**A**) The lysates of treated cells were investigated by Western blotting with anti-TRIM21, RABV P, and β-actin antibodies. (**B**) The total RNA of treated cells was harvested to determine the levels of RABV genomic RNA and the mRNA of TRIM21 by RT-qPCR. (**C**) The supernatants of infected cells were collected to determine the virus titers. (**D**) The infected cells were fixed and incubated with FITC-labeled anti-RABV-N antibodies (green) and DAPI (blue) to assess the RABV N protein by an immunofluorescence assay. Immunofluorescence images are magnified to 10*20. (**E**) We calculated the number of fluorescent spots in three different fields of view of the immunofluorescence experiments using ImageJ, and the results are expressed as the means ± SD; *, *p* < 0.05.

TRIM21 plays an important regulatory role in viral infection [33,34,35,36,37]. Having observed that TRIM21 is upregulated by RABV infection, we hypothesized that TRIM21 may be a crucial host factor in response to RABV infection. To test the hypothesis, small interfering RNA of TRIM21 (siTRIM21) and overexpression of TRIM21 were employed. After RABV infection, TRIM21 knockdown reduced the RABV P protein level (Figure 2A), genomic RNA level (Figure 2B), virus titers (Figure 2C), and N protein immunofluorescence of RABV (Figure 2D) at both 12 and 24 h post infection (hpi). Conversely, TRIM21 overexpression increased the RABV P protein level, genomic RNA level, virus titers, and N protein immunofluorescence of RABV at 12 and 24 hpi, as expected (Figure 2). Overexpression of TRIM21 promoted RABV replication and was dose-dependent. The results indicate that TRIM21 positively regulates the replication of RABV.

### 2.3. TRIM21 Promotes RABV by Increasing the Expression of Type-I Interferon

**Figure 3:**
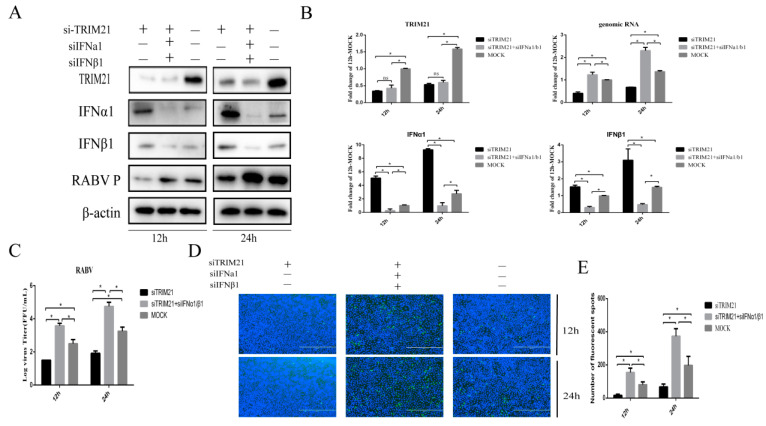
TRIM21 promotes RABV production by downregulating the expression of type I IFN. NA cells were transfected with TRIM21 or IFNα/IFNβ siRNA (siTRIM21, siIFNα, and siIFNβ) (1 μg) for 24 h followed by infection with RABV at an MOI of 0.5 for another 12 h and 24 h. siNC served as the control. (**A**) The total protein samples of treated cells were collected to determine the levels of TRIM21, IFNα, IFNβ, and RABV P by Western blotting. (**B**) The total RNA of treated cells was harvested to determine the levels of RABV genomic RNA and the mRNA of TRIM21, IFNα, and IFNβ by RT-qPCR. (**C**) The released virus titer from the differently treated cells was detected and illustrated using a TCID_50_ assay. (**D**) The treated cells were fixed, and an immunofluorescence assay was performed to detect the RABV N protein with FITC-labeled anti-RABV-N antibodies (green) and DAPI (blue). Immunofluorescence images are magnified to 10*20. (**E**) We calculated the number of fluorescent spots in three different fields of view of the immunofluorescence experiments using ImageJ, and the results are expressed as the means ± SD; *, *p* < 0.05; ns, non-significant.

Type I interferon plays a crucial role in the clearance of RABV during the infection [12,38]. Next, IFNα and IFNβ were assessed to investigate whether TRIM21 positively regulates the replication of RABV through the type I IFN signaling pathway. We found that knock-down of TRIM21 expression markedly diminished RABV replication at 12 and 24 hpi (Figure 3). Of interest, knockdown of IFNα and IFNβ (siIFNα and siIFNβ) significantly improved the RABV P protein level, which was attenuated by TRIM21 knockdown (Figure 3A) and increased genomic RNA (Figure 3B), virus titers (Figure 3C), and N protein immunofluorescence of RABV (Figure 3D) at 12 and 24 hpi. Meanwhile, the downregulation of TRIM21 also promoted the expression of IFNα1/β1 (Figure 3A,B). These observations indicate that TRIM21 promotes RABV replication by inhibiting the expression of type I IFN.

### 2.4. TRIM21 Regulates IFN Expression through IRF7 during RABV Infection

**Figure 4:**
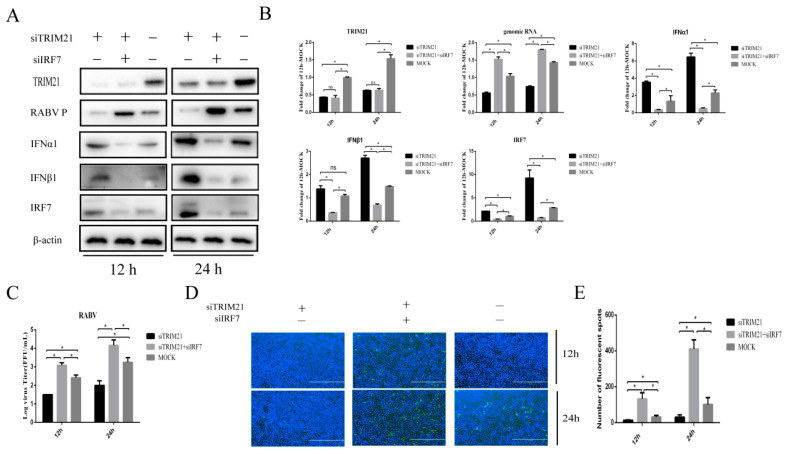
TRIM21 regulates type-I IFN through IRF7 during RABV infection. NA cells were transfected with TRIM21 or RIG-I siRNA (siRIG-I, siTRIM21) (1 μg) for 24 h followed by infection with RABV at an MOI of 0.5 for another 12 h and 24 h. NC served as the control. (**A**) The lysates of treated cells were assessed by Western blotting with anti- TRIM21, RABV P, IFNα1, IFNβ1, and IRF7 antibodies. (**B**) The total RNA of treated cells was harvested to determine the levels of RABV genomic RNA and the mRNA of TRIM21, IFNα, IFNβ, and IRF7 by RT-qPCR. (**C**) The supernatants of treated cells were collected to determine the virus titers. (**D**) The treated cells were fixed, and an immunofluorescence assay was performed to detect the RABV N protein with FITC-labeled anti-RABV-N antibodies (green) and DAPI (blue). Immunofluorescence images are magnified to 10*20. (**E**) We calculated the number of fluorescent spots in three different fields of view of the immunofluorescence experiments using ImageJ, and the results are expressed as the means ± SD; *, *p* < 0.05; ns, non-significant.

IRF7 is known to play a crucial role in interferon induction during RABV infection [10]. Next, we wondered whether TRIM21-mediated type-I IFN expression in RABV infection depends on IRF7. For this, TRIM21 and IRF7 were knocked down (siTRIM21, siIRF7) simultaneously. Then, the cells were infected with RABV for 12 h or 24 h, and the expression of IFNα/β was determined. In line with the results shown above, knockdown of TRIM21 increased the protein levels of IFNα and IFNβ (Figure 4A,B). As expected, knockdown of IRF7 attenuated the TRIM21 knockdown-induced expression of IFNα and IFNβ (Figure 4A,B). The results suggest that TRIM21 downregulates the expression of type I IFN through IRF7 in RABV infection. We next determined whether TRIM21 regulates RABV replication truly via IRF7I. The results showed that knockdown of IRF7 remarkably attenuated the TRIM21 knockdown-mediated reduction of the RABV P protein level (Figure 4A), genomic RNA level (Figure 4B), virus titers (Figure 4C), and N protein immunofluorescence of RABV (Figure 4D). Collectively, our findings support that TRIM21 regulates RABV replication through the IRF7-I–IFN axis [39,40,41].

### 2.5. TRIM21 Ubiquitination Degrades IRF7

**Figure 5:**
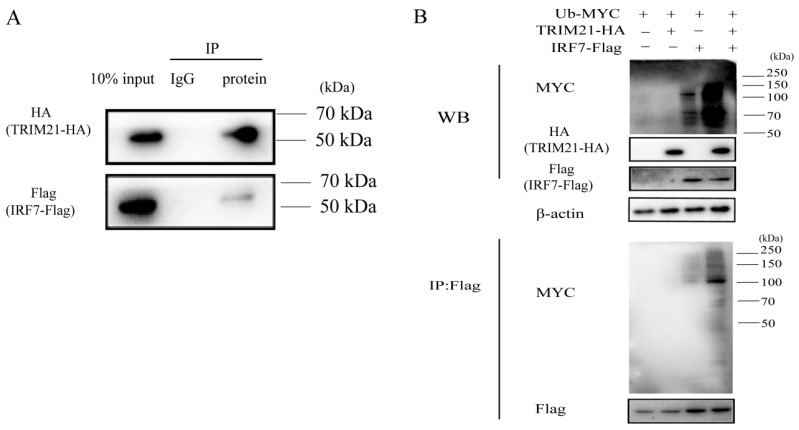
TRIM21 ubiquitinates IRF7 through direct interaction. (**A**) TRIM21 directly interacted with IRF7. HEK 293T cells were infected with an HA-tagged plasmid expressing TRIM21 and a Flag-tagged plasmid expressing IRF7 for 36 h. The whole cell lysates were incubated with an anti-Flag antibody or IgG conjugated magnetic beads at 4 °C for 10 h. The whole cell lysates (10%) and IP products were analyzed by Western blotting with an anti-HA antibody and anti-Flag antibody. (**B**) IRF7 was ubiquitinated by TRIM21. HEK 293T cells were infected with a Myc-tagged plasmid expressing ubiquitin (Ub), HA-tagged plasmid expressing TRIM21, and flag-tagged plasmid expressing IRF7 for 36 h. The whole cell lysates were analyzed by Western blotting with an anti-Ub antibody, anti-HA antibody, and anti-Flag antibody.

TRIM21 has been reported to be involved in the ubiquitination of IRF7 [32]. To further investigate how RABV triggers TRIM21 expression to regulate the IRF7–IFN axis, we first detected the ubiquitination of IRF7 mediated by TRIM21. As shown in Figure 5A, TRIM21 interacted with IRF7. TRIM21 overexpression increased the ubiquitination of IRF7 (Figure 5B), which agrees with the previous findings [36,37]. The results indicated that TRIM21 regulates RABV replication by ubiquitinating and degrading IRF7.

## 3. Discussion

Without proper treatment, the mortality rate from rabies can reach 100%. Therefore, it is important to understand the mechanism of RABV infection, particularly the host factors that participate in RABV infection. In this study, we found that RABV infection significantly upregulated the expression of TRIM21. Interestingly, TRIM21 was found to facilitate RABV infection. Studies have shown that numerous TRIM family proteins display antiviral activity despite many viral infections enhancing their expression. TRIM22 expression is upregulated under influenza A virus (IAV) and hepatitis C virus infections, and this protein inhibits the replication of these viruses [42,43]. TRIM32 expression is upregulated during a white spot syndrome virus infection, and the protein suppresses the activity of the virus. Ref. [44] Furthermore, the expression of TRIM25 is upregulated after infection with the infectious bursal disease virus (IBDV), which in turn inhibits the replication of the virus [45,46]. Herein, we found that RABV infection also upregulates the expression of TRIM21, but unlike the mentioned viruses, it promotes the replication of RABV. Studies have shown that TRIM21 promotes the replication of IAV through ubiquitination and degradation of IRF7 [32]. However, the replication regulation mechanism of TRIM21 differs among viruses. For instance, in vitro and in vivo studies have shown that TRIM21 expression is upregulated during infection with the porcine reproductive and respiratory syndrome virus (PRRSV), significantly inhibiting the replication of this virus [47]. In this study, we found that exogenous overexpression of TRIM21 significantly promoted RABV replication. Our findings suggest that TRIM21 is a novel host factor that promotes RABV replication, and thus, further investigation is needed to validate our finding.

Host antiviral activity involves either inhibition of the viral replication by restricting binding, entry, synthesis, assemble, and budding of the virus or clearing the virus using the innate immune response. TRIM21 regulates the innate immune response under viral infection through several mechanisms [48,49,50,51,52]. In our study, we found that overexpression of TRIM21 downregulated the secretion of IFNα and IFNβ levels in RABV infection, which subsequently promoted the replication of the virus. This was supported by the observation that the knockdown of IFNα and IFNβ substantially increased the production of RABV in NA cells, consistent with a previous study [53].

Viruses and their products can be recognized by several PRRs. The recognition induces the expression of IFN-I genes, activating the cellular antiviral response. Different pathways downstream of PRRs, including TLRs, RLRs, and DNA sensors, can transduce signals that converge on several key molecules, such as IRF family members. In most cases, IRF3 and IRF7 are required as base IRFs. IRF3 initiates the initial transcription of IFN-I and IRF7, and it is important for signal amplification of IFN-I transcription [54,55,56]. Although IRF7 is initially expressed at low levels, the formation of heterodimers between IRF3 and IRF7 is thought to be critical for the long-term production of IFN-I. Positive feedback regulation of IRF7 contributes to the host’s regulation of interferon expression in the late stage of viral infection [19,57]. A previous study showed that TRIM21 suppresses the antiviral responses during IAV infection by targeting IRF7 [32], which is consistent with our results in which we found that TRIM21 interacted with and degraded IRF7 during RABV infection through ubiquitination to inhibit the downstream IFN-I pathway. Thus, TRIM21 mediates RABV replication through the IRF7–IFN axis. Our study uncovered a novel mechanism by which type I interferons mediate RABV activity.

Studies have shown that FADD can form a complex with TRIM21 to jointly regulate the ubiquitination of IRF7 [30]. After IAV infection, N-myc and STAT interactor (NMI) can interact with IRF7. Furthermore, NMI promotes ubiquitination and proteasome-dependent degradation of IRF7 by recruiting the E3 ubiquitin ligase TRIM21 (triple motif-containing 21) to limit IAV-triggered innate immunity [22]. Therefore, there are still many unknowns about the regulatory mechanism of TRIM21 on IRF7 during RABV infection that need further exploration.

**Figure 6:**
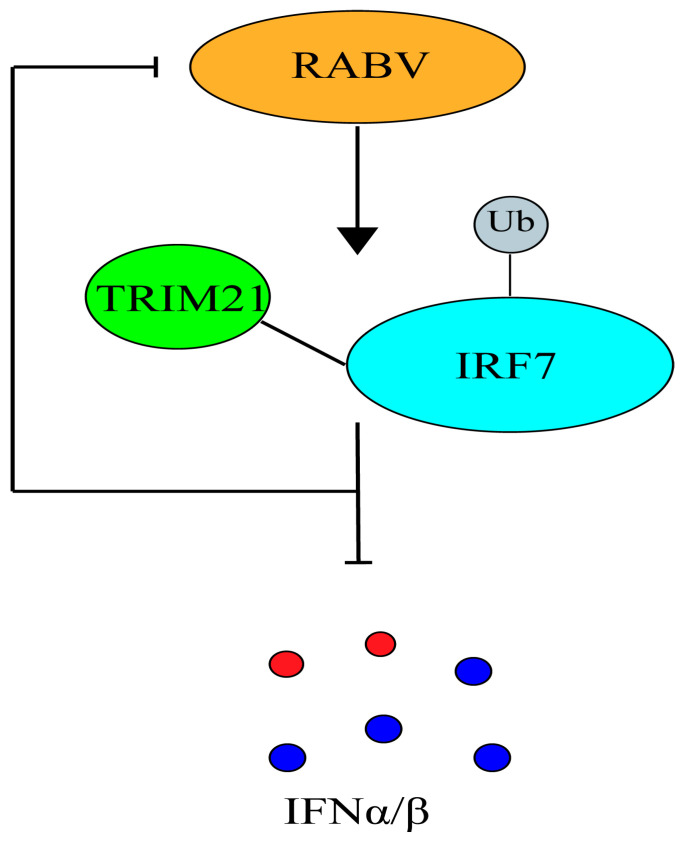
A model in which TRIM21 promotes RABV replication through inhibition of the IRF7–IFN axis.

In conclusion, this study identified TRIM21 as a novel host factor that regulates RABV infection (Figure 6). This study provided evidence of how TRIM21 regulates RABV replication. Generally, these findings deepen our understanding of the mechanism by which type I IFN regulates RABV infection and replication in the body. The findings of this study could be applied to developing new strategies for treating or managing rabies.

## 4. Materials and Methods

### 4.1. Cells and Viruses

NA cells (Wuhan Institute of Biological Products, Wuhan, China) were cultured in RPMI 1640 medium (Gibco) with 10% fetal bovine serum (FBS) (Gibco). HEK 293T cells were cultured in DMEM medium with 10% FBS. RABV fixed HEP-Flury strain (a gift from Dr. Kongwang He, Jiangsu Academy of Agricultural Sciences, Nanjing, China) was propagated in NA cells.

### 4.2. Plasmids, siRNAs, and Antibodies

HA-tagged pCAGGS (pCAGGS-HA), flag-tagged pCAGGS (pCAGGS-flag), and Myc-tagged ubiquitin (pcDNA3.1-Myc-Ub) plasmids were purchased from Wuhan Miaoling Biotechnology (Wuhan, China). The *TRIM25* gene from NA cells was cloned into pCAGGS-HA using the primers: TRIM21-F: 5′-AGCTCATCGATGGTACCCGGGCCATGTCACCCTCTACAACCTCAAAAAG-3′ and TRIM25-R: 5′-ATTAAGATCTGCTAGCTCGAGTCACATCTTTAGTGGACAGAGCTTTAG-3′ for overexpression of TRIM21. The *IRF7* gene from NA cells was cloned into pCAGGS-flag using the primers: IRF7-F: 5′-GCGAATTCATGGCTGAAGTGAGGGGGGTC-3′ and IRF7-R: 5′-GCCTCGAGTCAAGGCCACTGACCCAGGTC-3′ for overexpression of IRF7. The sequences of siRNA targeting interested proteins and the non-targeting control (NC) used in this study are shown in Table 1, which were synthesized by Sangon Biotech (Shanghai, China). Anti-TRIM21 antibody (#1E8B9) was purchased from Proteintech (Chicago, IL, USA); anti-IRF7 antibody (#CA2101) was purchased from Biohere (Kwangtung, Guangzhou, China); anti-HA antibody (sc-7392) and anti-Myc antibody (sc-40) from Santa Cruz Biotechnology (Dallas, TX, USA); anti-IFNα (ab191903) and anti-IFNβ (ab218229) from abcam (Cambridge, UK); and mouse IgG (A7028), anti-flag antibody (AF5051), and anti-β-actin antibody (AA128) from Beyotime Technology (Shanghai, China). Anti-RABV P antibody was prepared in our laboratory. FITC-labeled anti-RABV nucleoprotein (N) antibody was purchased from Fujirabio Diagnostics (Malvern, PA, USA).

### 4.3. RABV Titration

RABV titrations were conducted using direct fluorescent antibody assays (dFA) as previously described [58]. Briefly, NA cells cultured in 96-well plates were infected with 10-fold serial dilutions of RABV in RPMI 1640 medium. The cells were cultured at 37 °C for 2 days. The supernatants were then discarded, and the cells were fixed with 80% acetone for 30 min at −20 °C. Cells were washed and stained with FITC-labeled anti-RABV-N antibodies for 60 min at 37 °C. Antigen-positive foci were observed under a fluorescence microscope (AMG, Washington, DC, USA), and virus titers were calculated as focus forming units (FFU) per milliliter (FFU/mL) using the Karber method.

### 4.4. Immunofluorescence Assay

Treated NA cells were fixed with 80% acetone for 30 min at −20 °C. Cells were washed three times with PBS and stained with FITC-labeled anti-RABV-N antibodies for 60 min at 37 °C followed by the staining of DAPI. Antigen-positive foci were observed and photographed under a fluorescence microscope. ImageJ was used to calculate the number of fluorescent spots in three different fields of view and to perform a difference analysis.

### 4.5. Plasmid DNA and siRNA Transfection

A monolayer of NA cells or HEK 293T cells were transfected with recombinant plasmid or siRNA using Lipofectamine 3000 transfection reagent (Invitrogen, Carlsbad, CA, USA) according to the manufacturer’s instructions. An empty vector (pCAGGS-HA) was used as the control for plasmid transfection, while non-targeting control siRNA (NC) was used as the control for siRNA transfection. For more plasmids or siRNAs transfection, plasmid or siRNA was transfected at 12 h post the previous plasmid or siRNA transfection.

### 4.6. Co-Immunoprecipitation

Co-immunoprecipitation (Co-IP) was performed using the Pierce™ Crosslink Magnetic IP/Co-IP Kit (Thermo Fisher Scientific, Waltham, MA, USA) according to the manufacturer’s protocol. Briefly, following transfection with the indicated plasmids for 36 h, the whole cell lysates were prepared and used for immunoprecipitation with the indicated antibody-conjugated magnetic beads at 4 °C for 10 h. Normal IgG was used as a control. The IP products and whole cell lysates were analyzed by Western blotting (WB) with the indicated antibodies.

### 4.7. Western Blotting

Western blotting was performed as previously described [59]. Briefly, cell lysates or the IP products were separated by 12% sodium dodecyl sulfate-polyacrylamide gel electrophoresis and transferred to polyvinylidene difluoride membranes (Millipore, Bedford, USA). The membranes were then probed with the indicated antibodies. Protein bands were imaged using a Fine-do X6 Chemiluminescent Imaging System (Tanon, Shanghai, China).

### 4.8. Quantitative Real-Time PCR Analysis

Treated cells were harvested and total RNA was extracted using TRizol reagent (Magen, Guangzhou, China) according to the manufacturer’s protocol. Reverse transcription (RT) was performed using the Transcriptor First Strand cDNA Synthesis Kit (Vazyme Biotech, Nanjing, China). Each reaction was performed in triplicate using SYBR Green Master Mix (Vazyme Biotech). Quantitative real-time PCR (RT-qPCR) was performed in a CFX connect Real-Time System (Bio-Rad, Hercules, CA, USA). The levels of TRIM21 mRNA, IFNα mRNA, IFNβ mRNA, and RABV genomic RNA (gRNA) were normalized to glyceraldehyde-3-phosphate dehydrogenase (GAPDH). The primer sequences of TRIM21 were used: forward, 5′-GCTCCCTCATTTACACCTTCTCG-3′, reverse, 5′-GGCTCCTGACCATCACATCTTTAG-3′. The primer sequences of IFNα, IFNβ, gRNA, and GAPDH were described previously [60].

### 4.9. Statistical Analysis

Data were analyzed using GraphPad Prism 6 software (GraphPad Software, San Diego, CA, USA). Statistical significance was determined using a Student’s *t* test. *p* < 0.05 was considered statistically significant.

## Figures and Tables

**Table 1:** The siRNAs used in this study.

siRNA	Sense (5′–3′)	Antisense (5′–3′)
TRIM21-1	GGAGCCUAUGAGUAUCGAATT	UUCGAUACUCAUAGGCUCCTT
TRIM21-2	CCAAUAGACAUAUAGCCAATT	UUGUUAGGUGAGAAGUGGGTT
TRIM21-3	CCUGGACACGUUAGAUAUUTT	AAUAUCUAACGUGUCCAGGTT
IRF7-1	CCGCAUAAGGUGUACGAACUUTT	AAGUUCGUACACCUUAUGCGGTT
IRF7-2	CCUGGAAGCAUUUCGGUCGUATT	UACGACCGAAAUGCUUCCAGGTT
IRF7-3 IFNα-1 IFNα-2 IFNα-3	CUUCGACUUCAGCACUUUCUUTT GAGCCAGAUUAUCUCUUUCUATT CGUCAUUGAAUCACACCUGAUTT CAGUCAUUGAAAGCCUAGAAATT	AAGAAAGUGCUGAAGUCGAAGTT UAGAAAGAGAUAAUCUGGCUCTT AUCAGGUGUGAUUCAAUGACGTT UUUCUAGGCUUUCAAUGACUGTT
IFNβ-1	GCAGAAGAGUUACACUGCCUUTT	AAGGCAGUGUAACUCUUCUGCTT
IFNβ-2	AGCCCUCUCCAUCAACUAUAATT	UUAUAGUUGAUGGAGAGGGCUTT
IFNβ-3	GCUCUCCACUUGAAGAGCUAUTT	AUAGCUCUUCAAGUGGAGAGCTT
NC	UUCUCCGAACGUGUCACGUTT	ACGUGACACGUUCGGAGAATT

## Data Availability

The data presented in this study are available on request from the corresponding author. The data are not publicly available due to their containing information that could compromise the privacy of research participants.
